# Turning-ascending flight of a *Hipposideros pratti* bat

**DOI:** 10.1098/rsos.211788

**Published:** 2022-06-08

**Authors:** Aevelina Rahman, Peter Windes, Danesh Tafti

**Affiliations:** Department of Mechanical Engineering, Virginia Tech, 213E Goodwin Hall, 635 Prices Fork Road, Blacksburg, VA 24061, USA

**Keywords:** flapping flight, aerodynamics, manoeuvring bat flight, ascending and turning bat flight, manoeuvring techniques

## Abstract

Bats exhibit a high degree of agility and provide an excellent model system for bioinspired flight. The current study investigates an ascending right turn of a *Hipposideros pratti* bat and elucidates on the kinematic features and aerodynamic mechanisms used to effectuate the manoeuvre. The wing kinematics captured by a three-dimensional motion capture system is used as the boundary condition for the aerodynamic simulations featuring immersed boundary method. Results indicate that the bat uses roll and yaw rotations of the body to different extents synergistically to generate the centripetal force to initiate and sustain the turn. The turning moments are generated by drawing the wing inside the turn closer to the body, by introducing phase lags in force generation between the wings and redirecting force production to the outer part of the wing outside of the turn. Deceleration in flight speed, an increase in flapping frequency, shortening of the upstroke and thrust generation at the end of the upstroke were observed during the ascending manoeuvre. The bat consumes about 0.67 W power to execute the turning-ascending manoeuvre, which is approximately two times the power consumed by similar bats during level flight. Upon comparison with a similar manoeuvre by a *Hipposideros armiger* bat (Windes *et al*. 2020 *Bioinspir. Biomim*. **16**, abb78d. (doi:10.1088/1748-3190/abb78d)), some commonalities, as well as differences, were observed in the detailed wing kinematics and aerodynamics.

## 1. Introduction

Bats exhibit excellence at manoeuvring due to their highly articulated skeletal structures and pliant wing membrane that provides flexibility and control over aerodynamic force generation [1]. They show agility in air which is an important element for survival for most flying species as it is critical for capturing prey, avoiding predators or navigating through cluttered habitats. The natural agility and manoeuvrability allow bats to rapidly initiate a turn, to execute a turn within a tight radius or to redirect its trajectory within a wing beat. These flight traits are consequential in bioinspired micro air vehicle (MAV) design, and to this purpose birds and insects have been studied extensively in attempts to emulate desirable flight capabilities like speed, efficiency, quietness, endurance, durability, gust tolerance, light weight, control mechanisms and agility [2–15]. Studies regarding bat flights have received less attention in the literature compared with insects [8,12,14,16] mainly due to the challenges associated with measuring the complex wing articulation during flight. However, with recent advances in motion capturing technology [17,18], quite a few bat flight studies have been carried out providing a compelling model for highly agile and manoeuvrable MAV designs.

A significant portion of prior bat flight aerodynamics research has primarily focused on different aspects of straight flight such as wing and bone structure [1,19–22], flight efficiency and performance [23–29], complexity in wing kinematics [30–32] and aerodynamics [33–41]. These studies have provided tremendous insight into wing structure and articulation during flight and its effect on aerodynamic force generation. Manoeuvring flight on the other hand, brings in elements in wing articulation and aerodynamics not present in straight flight which are very worthy of investigation in spite of the challenges associated with capturing the wing kinematics during such flight and its aerodynamic interpretation [42–46]. Norberg [42] studied two bat species performing a 180° roll manoeuvre and a sideslip with a single camera. Although there were no three-dimensional kinematic data generated, a common mechanism was observed for both manoeuvres with pronation of one wing and supination of the other. Multiple cameras were used by Aldridge [46] to record the flight of six different species in a flight tunnel. Three-dimensional reconstructions were done for several of them, which resulted in correlations between turning radius and various morphological parameters. Iriarte-Díaz & Swartz [44] investigated 90° turns of fruit bats in an L-shaped tunnel with three-dimensional motion capture and were able to reconstruct detailed kinematic data. They observed that the right turn manoeuvre was effected by a combined bank and yaw rotation of the body. They also reported various kinematic parameters throughout the duration of the turn which shed light on the details of the turn. Henningsson *et al*. [45] investigated side manoeuvres of brown long-eared bats using particle image velocimetry (PIV) and three-dimensional motion capture, which enabled a coupled analysis of kinematics and the resulting flow field. They observed most manoeuvres to be initiated during the upstroke rather than during the downstroke with the most common use of asymmetries in drag or thrust. They also reported the time histories of wing amplitude, lift, thrust, wing length, angle of attack and body orientation for several manoeuvres. Boerma *et al*. [47] investigated the recovery manoeuvre from disruptive forces like aerial stumbles for fruit bats and reported that the bats primarily responded by adjusting extension of wing joints, and recovered pre-disturbance body orientation and symmetrical wing motions very fast, over the course of just one wingbeat cycle. They also used a simplified dynamical model that showed that the inertial torques generated during recovery drives the observed body reorientation. The same group also investigated landing manoeuvres and linked roosting ecology with landing biomechanics [48]. These studies while providing important insights into manoeuvring flight, also highlight some of the inherent challenges. To start with, data collection on flight kinematics in a wind tunnel suppresses the bat's ability to perform manoeuvres with significant heading change. For example, the shallow turn investigated by Henningsson *et al*. [45] resulted in a heading change of only 4°. Although the static L-shaped flight tunnel used by Iriarte-Díaz & Swartz [44] allowed complex manoeuvres with a heading angle change of as large as 45°, it could not allow for the use of PIV flow measurements for aerodynamic analysis.

Using measured wing kinematic data as input boundary conditions to run detailed aerodynamic simulations provides a powerful option for establishing the causal relationship between wing motion and force generation to effect the manoeuvre. Thus, the current study follows a computational approach to deconstruct the detailed kinematic–aerodynamic nuances of a manoeuvring bat flight. This approach has been used in the literature mostly for level flight [31,35,39] and recently for manoeuvring flight by Windes *et al*. [49,50]. Using measured kinematics and aerodynamic simulations, Windes *et al*. [49] investigated a right ascending sweeping turn of a large insectivorous bat (*Hipposideros armiger*) and found simultaneous and synergistic banking and yawing to be the fundamental turning mechanism. Later, an analysis of a U-turn of the same bat revealed that active control of the velocity along with the body rotations allows the bat to achieve the centripetal force for the 180° turn [50].

The current study is motivated by the desire to investigate a specific manoeuvre in a different bat species than done in previous works. In this paper, we investigate a *Hipposideros Pratti* (*H. pratti*) or Pratt's roundleaf bat while executing an ascending right sweeping turn. Combined kinematic and aerodynamic studies on bat manoeuvres are rare in the literature and provide a much needed gateway for a deeper characterization of the relationship between wing motions and the ensuing aerodynamics, which is the dominant force-producing mechanism over the course of the manoeuvre. The primary focus is to elucidate on the following: mechanism used to generate force asymmetries; dominance of lift, thrust or drag asymmetries; difference in mechanism between the initiation and rest of the turn; role of body rotations; contribution of banking and yawing; the relative contribution of upstrokes and down strokes and the energy cost of achieving the manoeuvre. These are investigated by a combined effort of kinematic measurements and detailed computational analysis of the aerodynamic forces generated during the turn. The kinematic data are collected in a flight tunnel using a three-dimensional optical motion capture system [28,35,43] while the numerical simulations are run using the wing kinematic data as an input boundary condition to calculate the flow and pressure field around the manoeuvring bat's wings, giving access to accurate spatially and temporally resolved force data on the wing surface throughout the entirety of the manoeuvre. Aerodynamic forces and rotational moments are analysed in two main reference frames; the global coordinate system and a body-fixed local coordinate system. This framework allows for investigating the underlying mechanisms causing body rotations and turning forces.

A secondary aim of this paper is to compare the current flight characteristics with that of Windes *et al*. [49] in which a *H. armiger* bat performs a similar manoeuvre. This is done with several caveats: to have a living creature perform the exact same manoeuvre in all its attributes is highly unlikely; even within the same species different bats will exhibit different individual traits that will influence their kinematics; and the same bat may have variations in how it performs the same manoeuvre. One way to eliminate these variations would be to take the mean of several flight experiments performing the same manoeuvre by measuring only a few characteristic data points on the body and wings. While this method will give macro-flight kinematic parameters, it will not yield the detailed wing kinematics needed to elucidate on the aerodynamic forces produced by the wing articulation to effect the manoeuvre. In spite of these caveats, because there are only a handful of investigations in the literature on aerodynamic characterization of manoeuvring bats, any insights into similarities and differences between flights, however limited, will contribute to a more comprehensive understanding of bat flight.

## 2. Methods

### 2.1. Experimental set-up and motion capture

The bat used for the current study is an adult female Pratt's roundleaf bat (*H. pratti*) weighing 55 g. The animal was kept with a group of conspecifics in a controlled indoor environment designed to allow natural movement given its typical flight behaviour. Ethical procedures according to Virginia Tech's Institutional Animal Care and Use Committee (protocol number 15-067) were followed. Kinematic data were collected using an optical three-dimensional motion capture system put together inside a 1.2 × 1.2 × 5 m open-ended flight tunnel. The system comprises 21 synchronized video cameras arranged in three rings located about 40 cm apart. The details of the camera specification and their arrangement is given in a prior work [35]. After being released, the bat flew without interruption through the tunnel and the camera arrays recorded the flight featuring different manoeuvres at 120 frames per second and in 1920 *×* 1080 pixel resolution. The camera array was calibrated using the Svoboda multi-camera self-calibration method [51].

The recorded flight path ([52] (Maneuvering_flight_trajectory.mp4)) consists of three full wingbeat cycles and an extra half-cycle at the end, i.e. 3.5 wingbeat cycles, captured over 55 video frames during which the bat executes an ascending right turn with deceleration in the flight direction similar to Windes *et al*. [49]. Each cycle is defined to start with an upstroke and end with a downstroke. The last half-cycle (upstroke) is included in spite of the second half of the cycle (downstroke) being incomplete as the bat continues to ascend outside the range of the motion capture system. After the last upstroke that was analysed in the current paper, the bat completes two full flaps before perching on the ceiling of the tunnel.

In order to track the wing motion, about 150 small white circular markers made of medical tape were set to the bat's wings to capture the detailed spatio-temporal kinematic features of the wing as it effectuates the manoeuvre. Stereo triangulation was performed for the 55 frames of the current flight using a custom Matlab code to achieve a total of (150 points) *×* (55 frames) ∼ 8250 points in three-dimensional space. In the event of spatial or temporal occlusions among those points, a temporal spline curve and a spatial implicit surface reconstruction [31] were used to fill in the missing data. Afterwards, a three-dimensional reconstruction was done using a Matlab code where a semi-automated technique was used to define point correspondences between frames, the details of which are described in previous works ([31,35,49,52] (Raw data for an ascending right turn of *Hipposiderous Pratti*)).

### 2.2. Reference frames

For kinematic and aerodynamic analysis, two reference frames are defined initially. The global or ground reference frame (*x_g_*, *y_g_*, *z_g_*) is a fixed, inertial coordinate system with *x_g_* directed along the longitudinal tunnel axis, *z_g_* directed upward opposing gravity and *y_g_* directed normal to both *x_g_* and *z_g_*.

**Figure 1.**
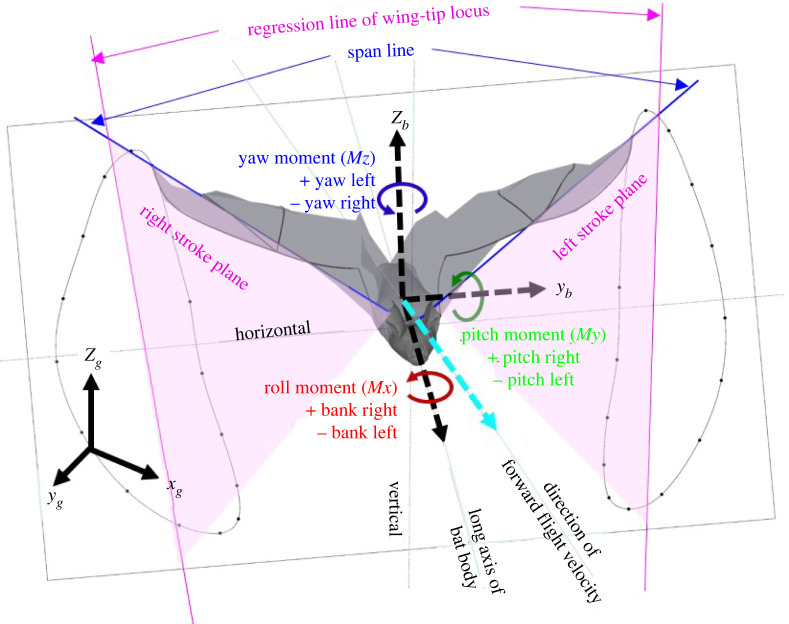
Definition of the global, body-fixed and velocity-based coordinate system with bat body orientation at an arbitrary point during flight.

The local or body-fixed reference frame (*x_b_*, *y_b_*, *z_b_*) is defined based on the instantaneous orientation of the bat's body and changes with time. The non-inertial body-fixed reference frame moves with the bat as it flies along; one such instance is shown in figure 1. The origin is set at the centre of mass (COM) of the bat body. The +ve *y_b_* vector points laterally toward the left wing while the +ve *x_b_* points along the axis of the bat body with perpendicularity enforced between the basis vectors *x_b_* and *y_b_*. Lastly, the *z_b_* upward pointing vector is found by taking a right-handed cross-product between *x_b_* and *y_b_*. Detailed definition and description of the three basis vectors in the body-fixed coordinate system is presented in Windes *et al*. [49]. The stroke plane, local aerodynamic forces and moments are all calculated using the instantaneous body-fixed coordinate system.

Another local coordinate system aligned with the velocity vector is also defined, as the long axis of the body might not always be aligned with the velocity vector of a manoeuvring trajectory. For example, in the current flight, the long axis of the body mostly lies inside the trajectory of the turn. The velocity-based reference frame (*x_v_* , *y_v_* , *z_v_*) is defined based on the instantaneous velocity vector which changes with time. Figure 1 shows such an instantaneous velocity vector in cyan which is also the direction of the +ve *x_v_* vector. The +ve *y_v_* vector again points laterally towards the left wing with orthogonality enforced with the *x_v_*-direction. The +ve *z_v_* vector points upwards and is found by taking a right-handed cross-product between *x_v_* and *y_v_*.

Figure 1 also defines the span (blue) as the line connecting the shoulder to the wingtip. The wingtip loci for the right and left wings are shown for a wingbeat cycle with two regression lines (pink) fitted through them. These regression lines along with a fixed root at the two sides of the wing make up the respective stroke planes shown in pale pink. Euler rotations about the body fixed *x_b_*, *y_b_* and *z_b_* axes are defined, respectively, as roll, pitch and yaw with right-roll, left-yaw and pitch-down as positive. The rotations apply in an orderly manner with yaw first, then pitch and then roll. The negative of the pitch angle is presented later in the paper as the elevation angle to denote pitch-up by a positive value.

### 2.3. Aerodynamic analysis

Following the kinematic data collection and pre-processing, the in-house incompressible Navier–Stokes solver, GenIDLEST [53] was used to simulate the aerodynamic flow around the bat during the manoeuvring flight. The immersed boundary method (IBM) is used to resolve the wing motion which is represented by a triangulated surface mesh immersed in a volumetric mesh. The location and spatial orientation of the surface mesh is advanced in time based on the wing kinematics ([52] (ctr_pts.ucd’, ‘surfgrid.s001’, ‘splines.dat’, ‘ibm_movement_bat.f90)). No slip boundary conditions are enforced on the wing surface.

**Figure 2.**
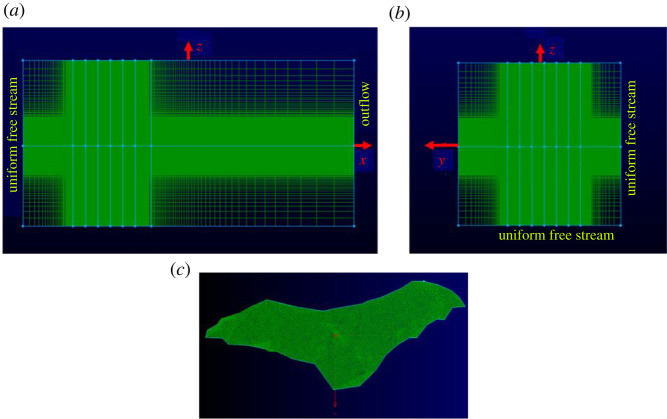
Schematic of computational grid (*a*) front view of background grid (38.2 million cells), (*b*) side view of background grid and (*c*) bat surface grid (42 000 surface elements).

The in-house Navier–Stokes solver has been used and validated for a diverse field of applications like bio-locomotion [54], bio-fluid mechanics [55], multiphase fluid-particulate system [56], turbo-machinery [57,58], heat transfer augmentation [59], etc. The IBM formulation specifically has been validated and applied to many different geometries and flow conditions, (e.g. [60–62]). For further details about the computational set-up used for the current paper, please refer to prior work on bat flight aerodynamics [35,39,49,50]. The computational domain extends from 8 chord lengths upstream to 24 chord lengths downstream in the *x_g_*-direction with a cross-section of 16 × 16 chord lengths in the *y_g_*- and *z_g_*-directions, respectively, representing the tunnel cross-section. In order to reduce the computational complexity of having to resolve the flying bat which would require a very fine mesh throughout the computational domain, a moving reference frame is used to limit the movement of the bat in the computational domain. The moving reference frame follows the mean velocity of flight, 2.40 m s^−1^ in the *x_g_*-direction, 0.36 m s^−1^ in the *y_g_*-direction and 0.54 m s^−1^ in the *z_g_*-direction. Perturbations on the mean flight velocities are reflected in the kinematics of the bat wings. The perturbed wing motion is shown in an animation in the supplementary documents ([52] (animation1.avi)). After the completion of the simulations, the results are post-processed by adding back the moving reference frame velocity. Figure 2 shows the background and surface grid distribution.

### 2.4. Validation

**Figure 3.**
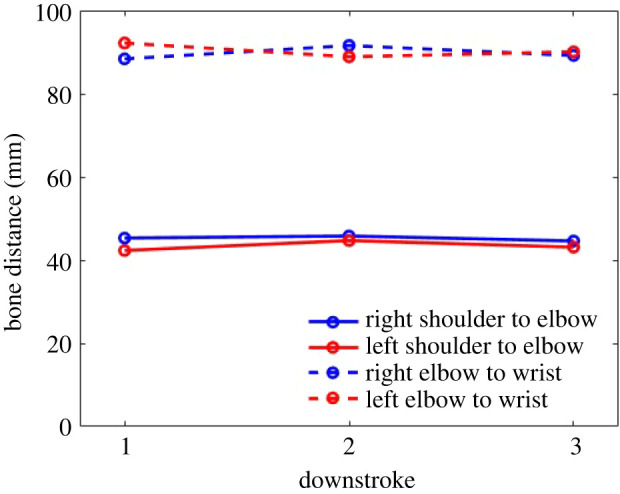
Comparison of right and left wing bone lengths at three time instances during downstrokes of the wing beat cycles.

In order to characterize the accuracy of the kinematic data from the motion capture methodology, the resulting lengths of two relatively rigid arm bones (bone 1: shoulder to elbow and bone 2: elbow to wrist) are compared at different time instances within the flight by using the derived spatial locations of shoulder, elbow and wrist markers. This is done for both the left and the right wing bones. The comparisons are done at the most outstretched frames of each downstroke. Figure 3 shows that the derived bone lengths from both wings are within 5% of each other over different cycles, validating the motion capture methodology and the extracted kinematic data from the raw measurements. Considering that the diameter of each white marker used was 3 mm and the bone lengths measured are approximately 50 mm (shoulder to elbow) and approximately 90 mm (elbow to wrist), the observed difference could very likely be due to the uncertainty associated with identifying the centre of each marker location. As will be shown later, the ability of the simulated aerodynamic forces to reproduce the experimental flight trajectory of the bat further validates the accuracy of the extracted kinematic data which is used as input to the aerodynamic simulations.

**Figure 4.**
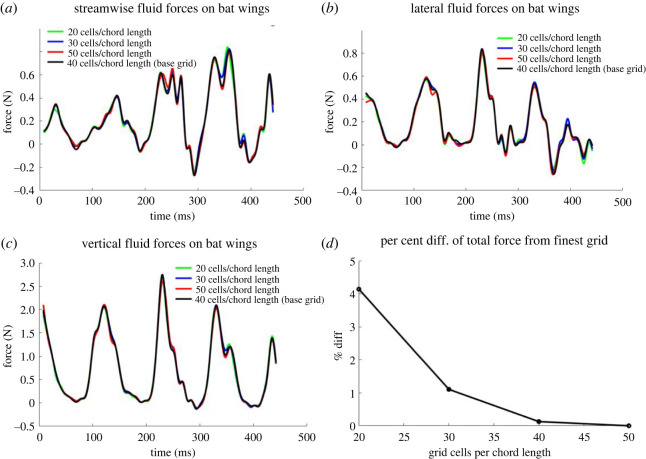
(*a*–*c*) Fluid forces in the global coordinate system on the bat wing on background grids of different resolution along with (*d*) percentage difference of net force from the finest grid.

To validate the aerodynamic analysis, four different grid sizes are tested for their ability to predict the time-dependent forces generated by the bat during the recorded flight. These are summarized in table 1. The coarsest background grid resolves the bat with 20 cells per wing chord length, whereas the finest grid uses up to 50 cells per chord length, with approximately 42 000 triangular surface elements (Δ ∼ 0.022 chord) defining the wing surface. The temporal evolution of generated forces in the *x_g_*-, *y_g_*- and *z_g_*-directions are presented in figure 4*a–c*. Some difference at force peaks and valleys where the temporal gradients are high for the two coarsest grids of 20 and 30 cells per chord length are evident. These differences are mitigated considerably as the grid is refined further to 40 cells per chord length. Figure 4*d* shows the percentage difference of the integrated net fluid force exerted over the full recorded flight compared with the finest grid of 50 cells per chord length. For the two coarsest grids the force varies by about 4% and 1%, respectively, while the difference is negligible when using the third (1/40 chord length grid spacing) grid. Based on this study, results from the 40 cells per chord length are presented in this paper.

**Table 1.** Summary of different background grids evaluated.

Cells per chord length/Δ*s*	Background grid size	Processors used
20/0.05	4.98 million	32
30/0.033	13.8 million	64
40 (base grid)/0.025	38.2 million	128
50/0.020	64.6 million	256

**Figure 5.**
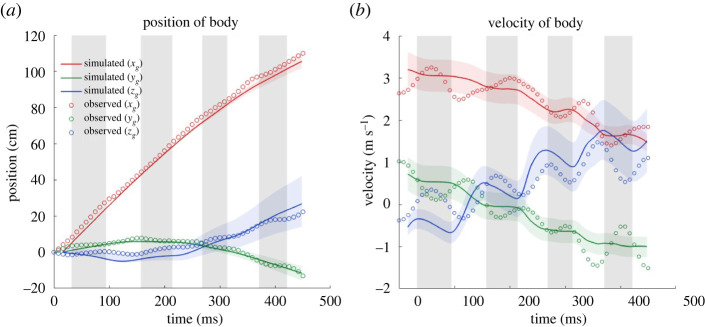
Comparison between the observed and predicted flight trajectory of the bat body in the global coordinate system; (*a*) position of the bat body and (*b*) velocity of the bat body; simulated velocities and positions are calculated from integrating the simulated force; grey shaded regions denote upstrokes while white denotes down strokes; red, green and blue shaded regions enveloping the predicted trajectory and velocity represents ±15% of the computed values from aerodynamic force, within which the observed position and velocity from kinematics lie.

To supplement the validation of the digitization of two-dimensional motion capture video frames to three-dimensional spatio-temporal data, and the grid independency study of the aerodynamic solver, additional validation is done by treating the bat as a simple lumped mass system and using the calculated aerodynamic forces ([52] (ibm_force_all.dat)) to predict its trajectory in comparison with the measured trajectory in the global reference frame. Note that this validation requires both the accurate representation of the actual wing kinematics as well as the accurate reproduction of forces driving the flight. The lumped mass dynamics analysis is based on Newton's second law, $d^{2}x/dt^{2}=F/m$. The bat mass is approximated as a point mass at the COM which is approximated by the body location and wing posture [63]. The net time-dependent forces are obtained from the numerical simulations. The velocity and position of the COM is then predicted by integrating the acceleration once and twice with respect to time. The comparison of the predicted path of the bat's body obtained from the aerodynamic simulation with the measured path of the bat body marker point is presented in figure 5.

Good comparison is obtained between the predicted and observed values of the body position and velocity in the *y_g_*- and *z_g_*-directions. The under-prediction of the velocity and consequently the position in the *x_g_*-direction indicates the under-prediction of *x_g_*-directional force by the simulation model. As pointed out by Windes *et al*. [49] and expressed in figure 5 by the shaded regions, even a small discrepancy in the predicted force will accumulate in time while calculating the velocity and position as they are calculated via temporal integration of acceleration. The shaded regions enveloping the predicted trajectory and velocity are representative of the observed values varying within ±15% of the predicted values. Another discrepancy, also observed by Windes *et al*. [49], is the relative insensitivity of the model to variations within a single wingbeat cycle. This trait is particularly characteristic of variations in the *x_g_*- and *y_g_*-directions and could be attributed to the simplicity of the lumped mass model, the much smaller aerodynamic forces produced in these directions compared with the *z_g_*-direction, and to inertial forces which are not included in the model. In spite of these differences, it is established that the overall trajectory over the recorded flight is reproduced with good accuracy, giving confidence in the fidelity of kinematic measurements and aerodynamic simulations.

## 3. Results and discussion

Of primary interest is relating the dynamics of the turning-ascending manoeuvre with the relevant wing kinematics. First, we introduce relevant morphological parameters of the bat and the flight trajectory followed by the aerodynamic analysis of the related wing kinematics. Wherever relevant we also compare and contrast the kinematic and aerodynamic traits of the flight of the *H. armiger* by Windes *et al*. [49].

### 3.1. Morphological parameters

The measured morphological parameters of the *H. pratti* in flight over the recorded 3.5 wing beat cycles are presented in table 2 along with equivalent data from Windes *et al*. [49] for a *H. armiger* bat for comparison.

**Table 2.** Morphological parameters.

	Mass (g)	Span (cm)	Wing area (cm^2^)	Planform area (cm^2^)	Chord (cm)	AR	Wing loading (N m^−2^)
Current flight	55	53	522 (mean = 398)	459	8.91	6.12	11.98
Windes *et al*. [49]	54.5	51	434	398	7.8	6.5	13.4

Definitions of the different parameters are as follows:

— *Wing area*. The wing area is the maximum total surface area during the downstroke (when the wing is fully stretched), averaged over several wingbeat cycles. The value in parenthesis indicates the averaged area over the entire up and downstrokes.
— *Planform area*. The planform area is the mean wing area during downstroke and upstroke projected onto the local body-fixed *x_b_*–*y_b_* plane, averaged over several wingbeat cycles.
— *Span*. The span is measured as the maximum wingtip to wingtip distance averaged over the wingbeat cycles.
— *Mean chord*. The mean chord of the bat wing is calculated by dividing the maximum planform area within a cycle by the span distance and averaged over the several wingbeat cycles.
— *Aspect ratio*. Aspect ratio (AR) is span squared over planform area
— *Wing loading*. Wing loading is weight over planform area.

The current bat exhibits a much larger mass and wingspan than some prior work on manoeuvring bat flight in the literature by Iriarte-Díaz & Swartz (mass approx. 33 g) [44], and Henningsson *et al*. (mass approx. 10 g) [45]. However, the morphological parameters of the current study are in the same range as that of Windes *et al*. [49,50] in spite of the bat being from a different species. The mass (1% heavier) and span (3% larger) are almost identical, but the *H. pratti* bat in the current study has a wing area larger by 20%, and thus a 15% larger planform area. This leads to a larger mean chord (14%), smaller AR (6%) and smaller wing loading (11%) compared with the bat in the study of Windes *et al*. [49].

### 3.2. General flight description

**Figure 6.**
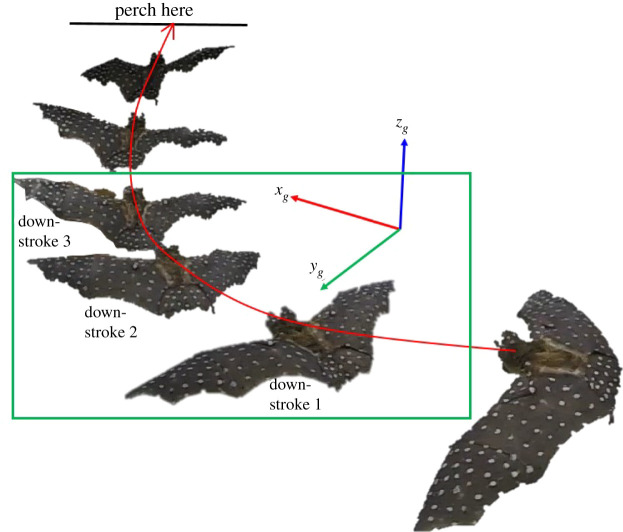
Flight trajectory (shown in red) with wing placement on six consecutive downstrokes. Current paper studies the three downstrokes (along with four upstrokes in between) shown in the green box. The bat perches on the ceiling of the flight tunnel on the third upstroke after downstroke 3.

In order to provide context for the presented results, this section describes the measured flight trajectory. Figure 6 shows the wing position at mid-downstroke of seven consecutive wingbeat cycles in the flight trajectory. Among them, three downstrokes (shown enclosed in the green box) and four upstrokes which constitute the right turn manoeuvre is studied in the current paper. Two wingbeat cycles later it performs an upside-down 180° somersault to perch on the ceiling of the tunnel. While the bat is still climbing, the preparation for the perch is expected to influence the flight during the latter stages of the recorded flight.

**Figure 7.**
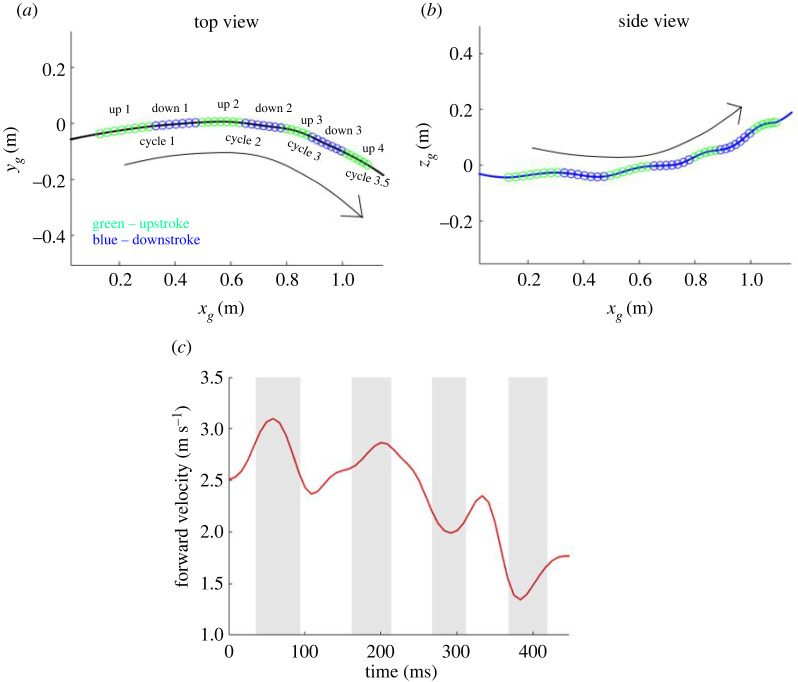
Flight trajectory (*a*,*b*) top and side views, green and blue dots depict individual frames of the video recording; (*c*) forward velocity in the local velocity-based coordinate system.

The analysed flight consists of 3.5 wingbeat cycles spread over 450 ms of flight time. Figure 7 provides planar top and side views of the flight trajectory identifying the upstrokes and downstrokes and the velocity magnitude along the flight trajectory. Among other things, figure 5*a* shows the details of the flight trajectory observed from the kinematic data (denoted by open circle symbols). Upstrokes and downstrokes are defined by taking the upmost and downmost positions of a mean of the left and right wing tips and wrist joints. Taking the starting position as the origin, the bat first veers to the left (maximum of 4 cm left from origin at 158 ms) as indicated by the lateral *y_g_*-displacement. It then makes a sustained right turn (maximum of 15 cm right from origin at 450 ms or at the end of the recorded flight). The entire flight is associated with a cumulative 46.4° change in bearing in the *x_g_*–*y_g_* plane. The manoeuvre is tightest at about 335 ms when the radius of curvature of the turn is smallest (=61.5 cm). During the course of the 110 cm recorded flight, the *x_g_*-directional velocity of the bat decreases from 2.95 to 2.15 m s^−1^. The initial mostly level flight (slight loss in altitude up to 150 ms), is followed by a gradual increase in elevation, becoming steeper at around 250 ms. During the recorded ascent, the bat gains an elevation of 19 cm. The maximum ascent angle is 26.9° during the last half-cycle. One interesting trait to note is that the dominant manoeuvres (right turn and ascension) in the lateral and vertical direction start at the same time (approx. 150 ms) as shown in figure 5*a*. This trait was also present in the manoeuvring flight of Windes *et al*. [49] indicating that, perhaps, a change in altitude is often accompanied by a turning manoeuvre or vice versa. Figure 7*c* shows the forward velocity of the bat in the local velocity-based coordinate system (as opposed to the global coordinate system used for figure 5*b*) in order to provide a clearer understanding of the effective velocities contributing to the current flight. It is seen that, during the climb and turn, the bat loses some of its forward momentum and decelerates from approximately 2.5 m s^−1^ to approximately 1.8 m s^−1^.

The quantitative flight parameters are noted in table 3 along with equivalent data from Windes *et al*. [49] for a *H. armiger* bat. The bat is flying much faster (about 45%) in the current flight and also decelerating more along its curved path when compared with the manoeuvring flight studied in Windes *et al*. [49]. The 2.64 m s^−1^ mean turning flight velocity in the present study is slightly higher but in the same range as the 2.0 m s^−1^ mean velocity observed by Iriate-Díaz *et al*. [44] and the wind tunnel velocity of 2.5 m s^−1^ used by Henningsson *et al*. [45].

**Table 3.** Comparison of flight parameters between current and a similar previous manoeuvre.

	Flight duration (s)	Total no. of full wingbeat cycles	Mean velocity (m s^−1^)	Mean acceleration (m s^−2^)	Wingbeat frequency (Hz)
Current flight	0.450	3.5	2.64	−1.78	9.08
Windes *et al*. [49]	0.558	5.0	1.81	−0.86	9.7

**Figure 8.**
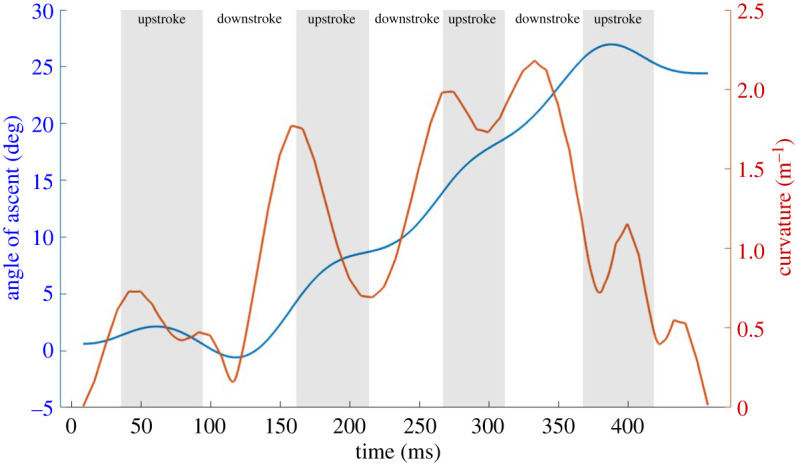
Angle of ascent and curvature over flight time; curvature is inverse of the radius of curvature (ROC), the tightest turn occurs when the ROC is smallest, or curvature is largest.

The average wingbeat frequency does not differ much between the two flights, although it is about 6% lower for the current flight. Analysing the angle of ascent and flight path curvature in figure 8 provides more perspective on the term ‘strongest manoeuvre’ in the vertical and lateral directions, respectively. In general, both the angle of ascent and curvature show an increasing trend during downstrokes and a decreasing or neutral trend during upstrokes, indicating that the majority of kinematic features fuelling both the upward and lateral manoeuvre take place during downstrokes. There is a clear synergy between the vertical ascent angle and lateral curvature. The angle of ascent has the steepest gradient during downstrokes followed by a lower rate of increase during upstrokes. Accordingly, the radius of curvature follows the same trend as the ascent angle, increasing and decreasing in sync with the rate of ascent. The curvature reaches a maximum at near mid-third downstroke indicating the tightest part of the turn in the lateral direction and drops off precipitously after that, indicating that the bat has straightened out of the turn. During this time, the bat keeps climbing but at a slower rate. The last part of the flight kinematics could be in response to the bat sensing the tunnel walls and preparing for the ceiling perch.

### 3.3. Flight aerodynamics

**Figure 9.**
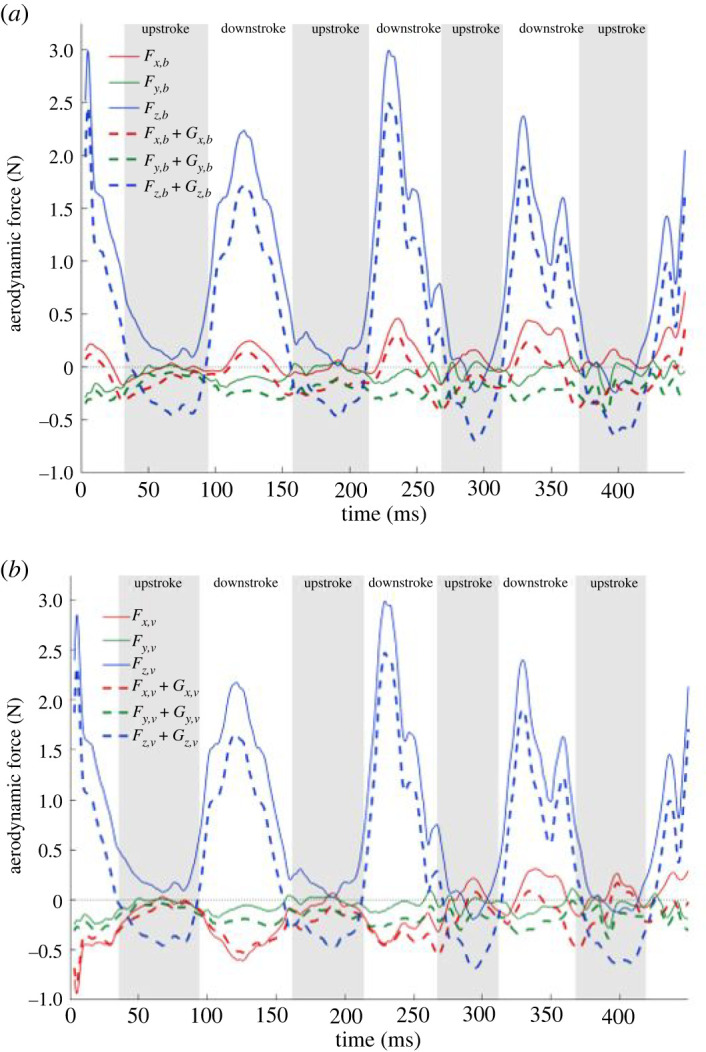
Forces in the (*a*) body and (*b*) velocity coordinate system with gravity components. *F* denotes aerodynamic forces and *G* denotes gravitational force components.

The integrated aerodynamic forces for the turning flight are first presented in the body-fixed coordinate system in figure 9*a* supplemented with the gravitational force components in the three directions. During straight, level, constant speed flight the aerodynamic forces supplemented with gravitational forces should balance out to be zero on a cycle averaged basis. However, for a turning, ascending and decelerating flight as the current one, significant forces are expected as shown by figure 9*a*. The *x_b_*-direction of the body frame is aligned with the direction of the longitudinal axis of the bat's body. Hence, the positive *x_b_*-component of the force is a major contributor to net thrust while the negative component to net drag. Positive peaks in $F_{x,b}\;$ (meaning instantaneous thrust is more than drag) are observed during the downstrokes. During the first two upstrokes, minimal net drag or a neutral value is observed. But during the major turning–climbing manoeuvre which starts at the second downstroke, small thrust peaks appear during third and fourth upstrokes to supplement the thrust generated during the downstrokes. Previously Johansson *et al*. [40] also reported thrust generation at the end of the upstroke and related it to effective pitch and yaw control. This feature was also observed by Viswanath *et al*. [64] during ascending flight of a fruit bat.

The *z_b_*-component of the force is the major contributor to the lift force and is substantially larger than the other two components in order to support the weight of the bat and to perform the climb. The body-coordinate *z*-directional force is maximum during the second downstroke to facilitate the steep climb in the subsequent cycles. The force generated during upstrokes is very small in magnitude, positive in the first two downstrokes and negative in the last two. The lateral force$\;(F_{y,b})$ in the body-fixed coordinate system remains near zero during the flight. On comparing $F_{x,b}\;$and $F_{z,b}$ with Windes *et al*. [49], the magnitude of $F_{x,b}\;$is comparatively smaller in the current flight while that of$\;F_{z,b}$ is comparatively larger. This could be because the rate of ascent of the current flight is higher than that of the *H. armiger* in Windes *et al*. [49]. Upon consideration of the gravitational force components, all the three directional body-fixed forces decrease or exhibit larger negative values.

At first glance, the overall positive force in the *x_b_*-direction seems counterintuitive to the overall deceleration observed in the current flight. However, as the direction of the long axis of the bat body is often different from the direction of the velocity vector, the force components calculated in the velocity-based coordinate system can provide a clearer relation with the observed bat motion. The velocity coordinate system is constructed by using the direction of the velocity vector as the *x_v_*-direction, followed by defining the *y_v_*- and *z_v_*-coordinate directions as outlined in §2.2 for the body-fixed coordinate system. Forces in the velocity-based coordinate system are plotted in figure 9*b*.

**Figure 10.**
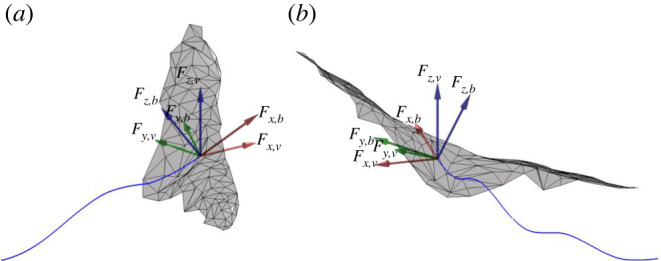
Contributions of forces in body-fixed coordinate system to forces in the velocity-based coordinate system; two different views (*a*,*b*) complement each other to show the contributions in the three different directions.

The biggest difference in the body-fixed versus velocity-based forces is evident in the forward direction. While the net $F_{x,b}$ force in the body frame is mostly positive, the forward component in the direction of velocity is mostly drag force. This is consistent with the observed motion of the bat slowing down. The manifestation of a net drag force $F_{x,v}$ in the velocity frame in spite of a net thrust force, $F_{x,b}$, in the body frame is explained by the relative orientation of the force components in the two coordinate systems shown in figure 10*a*. $F_{z,b},$by far the largest force in the body frame, has a large positive contribution to $F_{z,v}\;$in the velocity frame, but at the same time has a significant negative contribution to $F_{x,v}\;$to overwhelm the component of positive $F_{x,b}$. The small thrust peaks observed on both up- and downstrokes from the third cycle onwards result from the larger positive $F_{x,b}$ force. Figure 10*b* also shows that $F_{z,b}$ contributes a negative component to the velocity frame lateral force $F_{y,v}$, increasing its magnitude.

**Figure 11.**
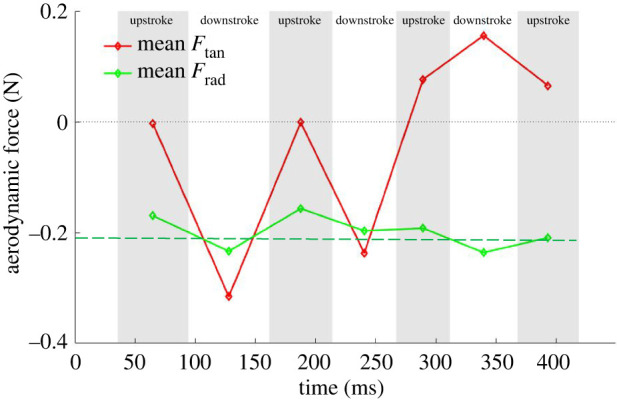
Half-cycle averaged tangential and radial force.

The inward pointing lateral force $\left(F_{y,v}+G_{y,v}\right)$ along the bat trajectory plays an instrumental role in a stable turn by balancing the centrifugal acceleration felt by the bat as it traverses the turn. From figure 11, the net lateral or radial force during the turn from 158 to 450 ms is calculated to be approximately 0.21 N (shown by the green dashed line). The centrifugal force generated is calculated from the mass, velocity and radius of turn in equation (3.1) and found to be 0.24 N. This balance between the forces acting on the turn radius assures a stable turn.

3.1 $$F_{\mathrm{rad}}=ma_{c}=\frac{mv_{\tan}^{2}}{r}\;\approx \;\frac{(0.055\;\mathrm{kg}){\left(\;2.27\;m\;s^{\;-1}\right)}^{2}}{\;1.15\;m}=0.24\;N$$

where *m*, total bat mass; *a_c_*, centrifugal acceleration; *v*_tan_, approximate flight velocity magnitude averaged over the flight time; *r*, approximate radius of curvature averaged over the flight time.

It is to note that the radial force shown in figures 9*b* and 11 results from the simulation, whereas the centrifugal force is obtained from the kinematic data; the close agreement between the two is yet another validation of the overall methodology. Discrepancies between the estimated (from kinematic data) and calculated (from aerodynamic simulations) values can result from the fact that the bat is not in a steady state, constant radius, constant velocity turn and is also gaining elevation.

### 3.4. Orientation of bat body

Another aspect of the turning mechanism comes from the orientation angles of the bat body and velocity. The direction of the net force vector is the most significant driver that allows the bat to follow a curved trajectory. This direction is predominantly controlled by the rotational orientation of the bat in space. The relative orientation of the body-fixed frame to the ground frame represents the angular rotation of the bat, described by the three Euler angles as the roll, pitch and yaw, as introduced in §2.2. When the bat changes its angular orientation, it changes the direction of the flight path through yaw and redirects the force so that a radial acceleration is achieved through roll.

As the direction of a flight does not always follow the body orientation, we define three additional angles based on the velocity coordinate system. Equivalent to the roll angle in the body-fixed coordinate, a bank angle is defined as the absolute angle between *y_v_*-axis and the horizontal. Also defined is a climb angle, which is the inclination of the *x_v_*-axis with the horizontal and a bearing angle as the angle between the initial and current flight direction projected on the horizontal *x_g_*–*y_g_* plane.

**Figure 12.**
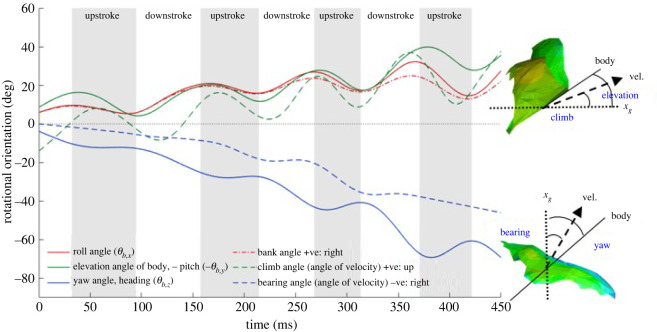
The angular orientation of the bat's body and velocity vector relative to the ground, slope of lines indicates angular velocity and concavity indicates angular acceleration. Schematics on the right show a representative (non-scaled) deviation of the longitudinal body axis and velocity vector and the resultant angles.

The Euler angles of the bat body rotation (yaw, pitch and roll) are shown in figure 12 along with the orientation of the velocity vector (bearing, climb and bank). The bank angle, although closely related to the roll angle, is not a Euler angle. As Euler rotations are applied sequentially, the roll angle is usually affected by the pitch and yaw angles. However, the bank angle is free from any such effect and can simply be calculated as the absolute angle between the instantaneous *y_v_*-axis and the horizontal. At small pitch angles, the roll and bank angles do not differ much as the effect of pitch on roll is minimal. This is evident at the start of the flight where pitch angles are smaller. However, towards the end of the flight, after the second cycle, the bat undergoes a steep climb, thus obtaining a significant pitch, which in turn affects the roll angle. Therefore, there is significant difference between the bank and roll angles towards the end of the flight. Figure 12 also shows the elevation angle, which is defined as a negative pitch angle for convenience. Positive values of the elevation angle represent an upward inclination of the bat body. The velocity counterpart of the elevation angle is the climb angle, which has been defined above. The last angle describing the flight velocity in figure 12 is the bearing angle, which is solely based on the flight direction, whereas the yaw angle is based on the body orientation only. The distinction between the body and velocity angles plays a fundamental role in explaining the manoeuvre mechanics below.

The body rotations allow the bat to redirect the net force vector so that a turning manoeuvre can be achieved. The most direct way to incline the force vector laterally to activate a turn is through rolling of the body and banking. A positive roll angle of around 7° from the start of the flight indicates that the bat is preparing for a right turn. During the flight, the roll angle increases gradually up to around 20° until the third cycle after which it grows rapidly to 37° towards the end. This is consistent with the curvature plot shown in figure 8 which indicates that the tightest portion of the turn starts at around the third cycle. Although roll is the most direct way to incline the net force vector in favour of a lateral turn, a combined yaw-roll action is more effective. Yaw allows the centre of the turn radius to be shifted back and thus produces a tighter turn. In the absence of yaw, only roll can result in a gradual turn, but not a tighter one. Similarly, only yaw without roll is not optimal for a tighter turn as the lift force remains vertical and does not impart any component towards the turn. Thus, only the significantly smaller thrust force imparts a radial component if there is no roll and only yaw. The current manoeuvre was achieved by a synergistic effect of roll and yaw. A negative yaw and a positive roll drives the bat to turn right as is seen for the current flight in figure 12. As yaw facilitates a rotated axis for subsequent roll to be performed around, it is a crucial component to initiate the turn. Therefore, the yaw angle in figure 12 shows a gradual increment in the first two cycles, when the lateral turn is not very tight. But when the third cycle starts, there is a steep increase in the yaw angle facilitating the tighter turn despite the bearing angle not following suit. The comparatively gradual behaviour of the bearing angle towards the end is consistent with the overhead flight trajectory of figure 7, showing that the bat bearing change is comparatively gradual throughout the entire flight time. Even after the collaborative effect of roll and yaw initiates the steep turn around the third cycle, the bearing angle does not align with the heading. This indicates that the body axis of the bat is always positioned inside the trajectory of the turn for the current flight.

The bat body pitches up during downstroke and pitches down during upstroke. As seen in figure 12, the increase in elevation during downstroke is larger than the decrease during upstroke allowing the bat to gain altitude with the steepest gain occurring during the third stroke. At the start, there is an approximately 37° difference between the elevation and climb angle, but during the manoeuvre the velocity is nearly aligned with body axis. By contrast, Windes *et al*. [49] reported a somewhat consistent difference of 20° between the body elevation angle and the velocity inclination angle for both the manoeuvre as well as straight flight for the *H. armiger*, implying that the noted difference between how a bat positions its body with respect to its trajectory could possibly be an individual trait of the bat.

### 3.5. Rotational moments

**Figure 13.**
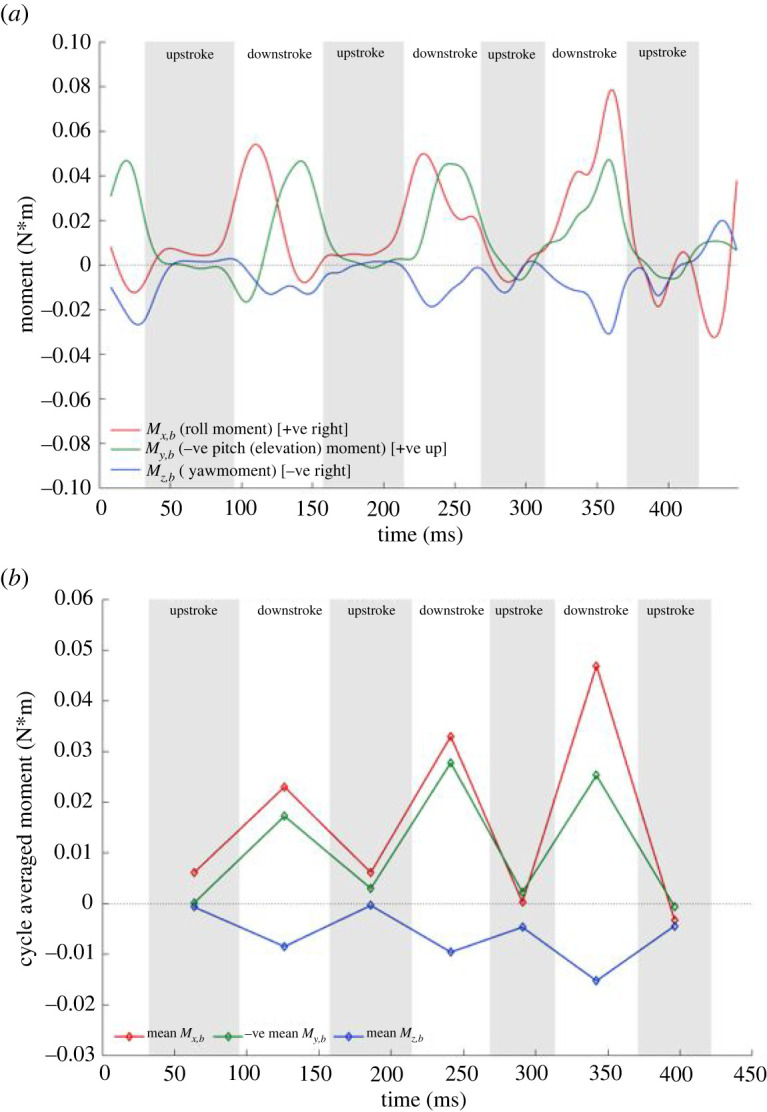
Body frame rotational moments relative to the centre of mass; (*a*) instantaneous and (*b*) cycle averaged.

Thus far, we have established that the bat turns by generating a lateral force which provides the needed centripetal acceleration. This lateral force is produced when the net force vector is reorientated by the combined effect of body yawing and banking. The moments created on the three axes of the body frame are yet another important aspect to explain the turning mechanism. The net rotational moment based on the bat's COM is estimated from the spatial distribution of the aerodynamic force on the wing surface. Figure 13 shows the roll, negative pitch or elevation and yaw moment created in the body-fixed coordinate system both in a transient (*a*) as well as half-cycle averaged (*b*) time axis. According to a right-handed system, a negative yaw and positive roll moment are necessary to make a right turn while a negative pitch (or positive elevation) moment takes the bat upwards as shown in figure 13.

The yaw and roll moments are the main drivers for the lateral turn. The cycle-averaged plot shows that the yaw moment experiences a gradual increment in the first two wingbeat cycles and then a significant increase starting from the third cycle. This is consistent with the increasing trend of the body yaw rotation seen in figure 12. The highest roll moment is also recorded towards the end of the flight where the tightest turn occurs. Towards the very end of the recorded flight, in the fourth upstroke and the subsequent downstroke, a correction can be seen in the form of a negative roll moment in the instantaneous plot, which is surmised to be preparation for the perch in the following cycles. The positive cycle averaged elevation moment fuels the overall upward motion of the bat. Within each wingbeat cycle, during the downstroke the predominantly positive elevation moment fuels the bat's upward pitch. Towards the end of every downstroke, the bat starts to pitch down, which persists to a steady and gradual decrease of the elevation moment during upstrokes. The highest rate of the elevation moment increment is seen between downstrokes 0 and 2 with the peak magnitude achieved just before the steepest ascent begins. It is noted that the bulk of the moments that effectuate the manoeuvre are generated during the downstroke when the aerodynamic forces are at their highest, whereas the upstroke plays a minor role.

**Figure 14.**
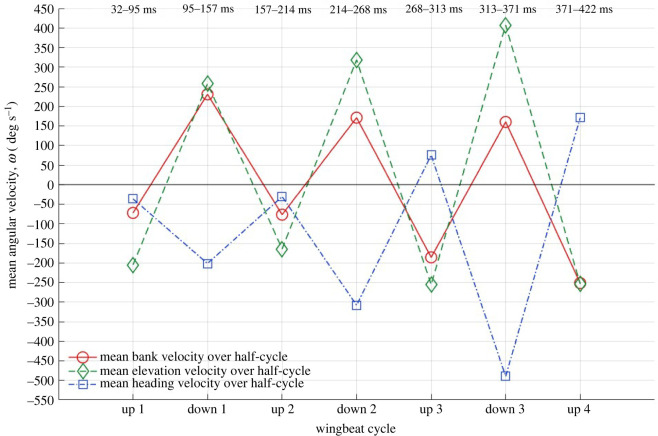
Half-cycle averaged angular velocities.

These aerodynamic moments give rise to angular acceleration or changes in angular velocities which are presented in figure 14 from the experimental kinematic data with a half-cycle averaged time axis for easier visualization. Angular acceleration or the change in angular velocity can be caused by aerodynamic moments, inertial moments or a combination of the two. The yaw or heading angular velocity is always net negative meaning that the bat nose is moving inward of the turn. In accordance with the yaw moment, the yaw or heading angular velocity increases to a maximum of 500 deg s^−1^ of yaw rotation during the initiation of the tightest turn (at around the third cycle). The angular bank velocity changes from net positive to slightly net negative towards the end of the flight in accordance with the corrective negative roll moment observed in preparation for the perch. The elevation angular velocity is overall positive and increases with the upward motion, peaking up to 400 deg s^−1^ of pitch rotation nearing the end of the flight. This is consistent with the steep ascent observed towards the end of the current flight.

### 3.6. Spatial distribution of aerodynamic forces and their effect on rotational moments and turning mechanism

**Figure 15.**
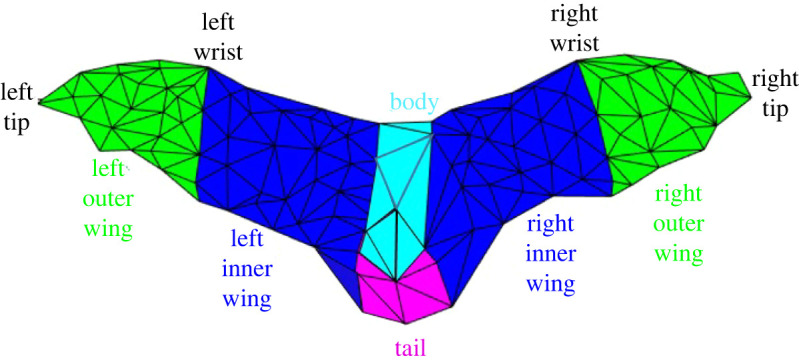
Spatial segmentation of the wing.

The body rotations and moments are created when the wing kinematics cause asymmetries between the forces generated by the left and right wings as well as between different regions of the wing surface (inner and outer). The different regions of the wing on which the force distribution and thus moment generation is analysed are specified in figure 15.

To better understand the effect of the spatial distribution of forces on moment generation, forces on inner and outer wings in the body-fixed local coordinate system are presented.

**Figure 16.**
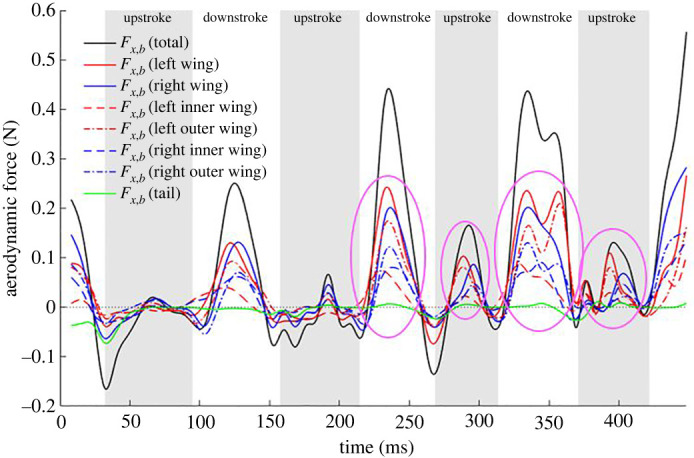
$F_{x,b}\;$ component of aerodynamic force broken down by different wing sections; pink circled regions encompass the asymmetries observed between the left and right wing.

Figure 16 shows the body-coordinate *x*-directional force acting on different regions of the wing. Imbalance in the thrust generated by the left and right wing is a major contributor to the yaw moment and body rotation. Starting from the second downstroke, three types of asymmetries are noted in the force distributions. The first is a phase lag between left and right wing, the second is the left wing producing a larger positive force than the right wing, and the third is the left outer wing consistently producing more force than its right counterpart. These trends are persistent during the second and third strokes and are marked by pink circled regions. The asymmetries contribute to a large extent to the negative yaw moment (rotates the nose into the turn) that allows the subsequent roll to be acted on an already rotated longitudinal axis. Similar observations were made by Windes *et al*. [49].

**Figure 17.**
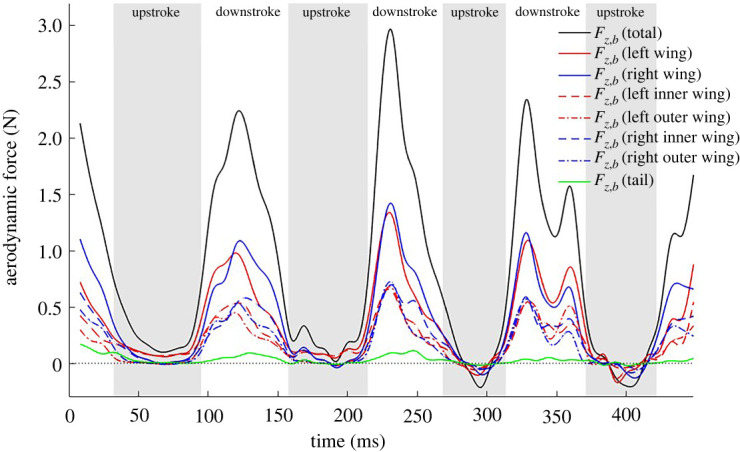
$F_{z,b}$ component of aerodynamic force broken down by different wing sections.

**Figure 18.**
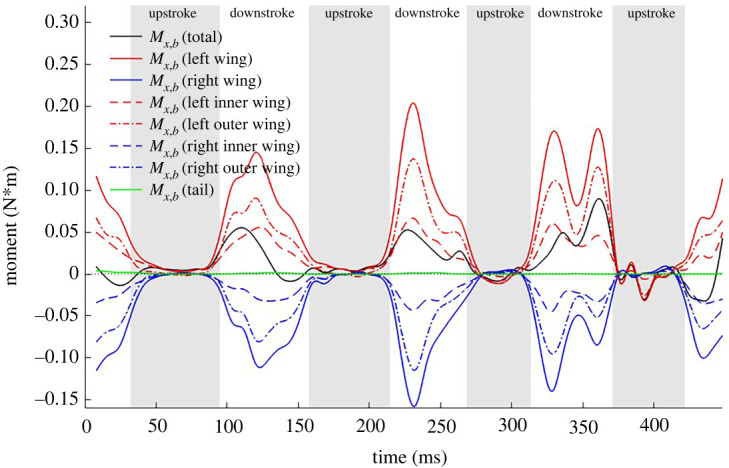
Roll moment generated by wings.

Figure 17 shows the body-coordinate *z*-directional force acting on different regions of the wings. An imbalance in the force generated by the left and right wing contributes to the roll moment in the body frame. $F_{z,b}\;$ has a slightly larger contribution from the right wing during downstrokes 1 and 2 and from the left wing during downstroke 3 and 4. Otherwise, the contribution of the left–right and the outer–inner wings show relative symmetry in the force generated in the body-coordinate system and cannot be clearly and consistently related to the generation of roll moments presented in figure 13. In this case, the bat effectuates the required roll moment by positioning the two wings differently and changing the moment arm between the left and right wing during the right-turn manoeuvre. This is reflected in the roll moments generated by the individual wings shown in figure 18. It will be shown in §3.8 that during the right turn, the right wing moves closer to the body, thus reducing the moment arm on which the aerodynamics forces act, thus effectuating a right roll.

### 3.7. Wing kinematic traits

In this section, we investigate different kinematic traits of the bat flight. We also focus on comparing these traits with the right ascending turn studied by Windes *et al*. [49]. In the full spectrum of manoeuvres that a living bat could possibly undertake, the manoeuvre studied in this paper and that by Windes *et al*. are quite similar. In both cases the bats, which morphologically are also quite similar, start out at near level flight and initiate an ascending right turn manoeuvre in a similar, if not exactly the same flight tunnel environment. During the 450 ms duration of the recorded flight, the bat ascends about 20 cm, while travelling a distance of 110 cm along the length of the tunnel and moves 17 cm laterally to the right. By contrast, the bat in [49] travels 87 cm along the length of the tunnel, ascending 18 cm with a lateral displacement of 32 cm during the recorded flight of 550 ms. While the rate of lateral movement between the two bats is approximately the same, the current bat is more aggressive in the rate of ascent and travels faster in forward flight. Given these similarities and differences we seek to identify any general traits in their kinematic signatures, noting that in the current study the bat perches on the ceiling two flaps past the end of the recorded flight, and this action is bound to impact the kinematics towards the end of the recorded sequence.

**Figure 19.**
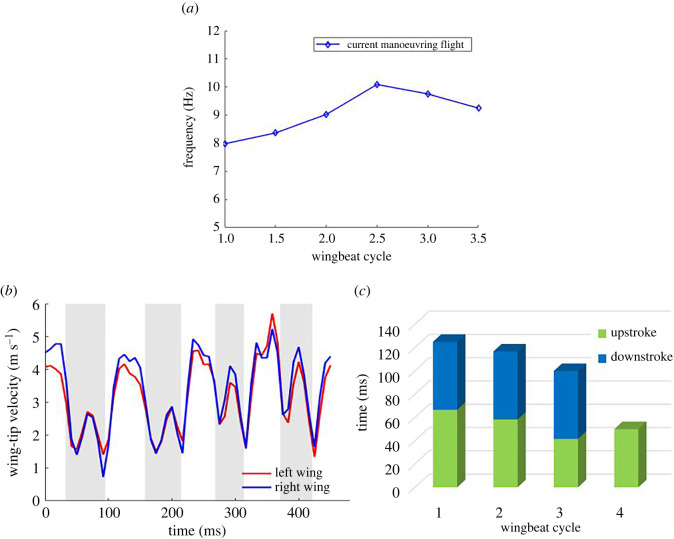
(*a*) Temporal trend in wing beat frequency shown at each half-cycle; (*b*) tip velocity for the left and right wing; (*c*) time spent in upstrokes and downstrokes in consecutive wingbeat cycles. The fourth upstroke was not analysed in the current study.

Figure 19*a* shows the flapping frequencies for different wingbeat cycles. It is notable that the frequency increases at the initiation of and during the manoeuvre. This occurs at the start of the second cycle in the current flight during which the bat starts ascending. Thus, an increase in flapping frequency can be categorized as one of the mechanisms bats use to increase the power needed to effectuate the manoeuvre but may not be singular to this particular manoeuvre. A similar observation was made in Windes *et al*. [49]. There lies an aerodynamic limit to the maximum amount of lift that can be generated in a single stroke. Thus, to get additional lift for ascent a bat needs to execute more downstrokes per unit time (i.e. increase flapping frequency). This is done by increasing the downstroke velocity and by shortening the duration of the upstroke. Figure 19*b* shows that the tip velocity of the current bat for both wings increases, peaking at the third cycle when the maximum frequency is observed (figure 19*b*). Additionally, the bat also spends less time on upstrokes (again lowest in the third cycle) as shown in figure 19*c* by increasing the upstroke wing velocity more than the corresponding increase in downstroke velocity. This allows the downstrokes to be longer to accommodate the excess lift generation needed for the ascent.

The wing kinematics define the shape and orientation of the bat wings over the duration of flight. The mechanisms by which bats generate lift to stay aloft and thrust to propel forward, as well as the forces and moments to manoeuvre, are controlled by the wing kinematics. In order to provide a holistic view of the kinematic data and identify specific manoeuvring traits, several parameters which characterize important aspects of wing motion are presented. Before going into the detailed kinematic markers, it is worth mentioning that a consistent difference observed between the current flight and that of Windes *et al*. [49], was the positioning of the body with respect to the velocity vector. Figure 12 shows that the bearing angle has a larger negative value than the heading angle, implying that the body long axis is oriented inside the trajectory of the turn. In the previous sweeping turn, during initiation the body was positioned outside the turn but quickly aligned with the velocity vector during the rest of the sweeping turn. This basic difference could instigate differing kinematic mechanisms to effectuate a similar manoeuvre.

### 3.8. Orientation of stroke planes and associated angles

**Figure 20.**
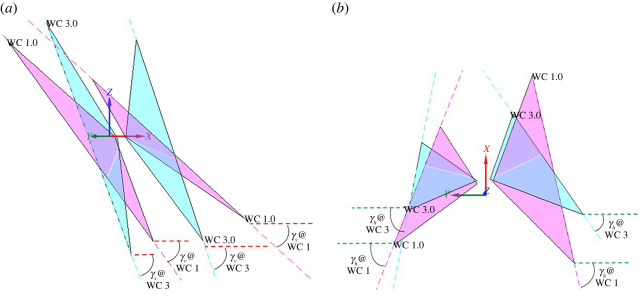
Stroke planes for first (magenta) and third (cyan) wingbeat cycles for the left and right wings; WC, wingbeat cycle; the dashed lines show the regression line of the wingtip loci for respective cycles. (*a*,*b*) show different views of the stroke planes for better perspective of the vertical (*γ_v_*) and horizontal (*γ_h_*) stroke plane angles.

Stroke plane is defined in §2.2 (schematic in figure 1) as the plane formed by the regression line through the wingtip loci of a complete wingbeat cycle and the root of the wing. Two separate stroke planes are calculated for the right and left wings. A new stroke plane is calculated at each half wingbeat cycle, e.g. a stroke plane at wingbeat cycle 1 is calculated using the first upstroke + first downstroke, cycle 1.5 is calculated using first downstroke + second upstroke, and so on. Figure 20 shows the different stroke planes calculated for the first and third wingbeat cycles for the left and right wings.

In spite of the fact that bat wings are not rigid planar surfaces but highly deformable membrane wings, the orientations of these planes are used to identify macro adjustments that the bat makes during manoeuvres. The orientation of the stroke plane is described by the horizontal $(\gamma_{h})\;$and vertical $(\gamma_{v})\;$stroke plane angles. The horizontal stroke plane angle $\gamma_{h}$ is obtained by projecting the wingtip loci regression line onto the body-fixed *x_b_*–*y_b_* plane. The angle between the projected regression line and the *y_b_*-axis is taken as the horizontal stroke plane angle which represents the in-out or lateral movement of the wingtip in relation to the body. The projection of the regression line onto the body-fixed vertical (*x_b_*–*z_b_*) plane defines the vertical stroke plane angle $\gamma_{v}$ as the angle between the projected line and the *x_b_*-axis. It can vary from 0° to 90° with higher angles facilitating more thrust and lower angles more lift. It signifies the forward–backward movement of the wingtip. Symmetry between the left and right stroke plane angles is expected during straight flight, while asymmetries initiate the imbalance in force usually seen in manoeuvring flights.

**Figure 21.**
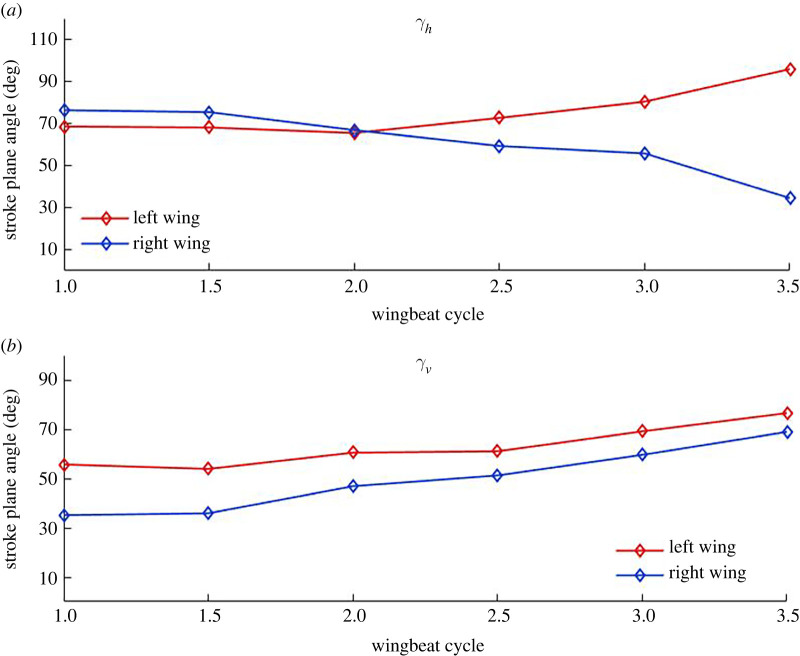
The vertical (*γ_v_*) and horizontal (*γ_h_*) stroke plane angles for the right and left wings.

The horizontal and vertical stroke plane angles for the current manoeuvring flight are presented in figure 21. The horizontal angle, $\gamma_{h}$ exhibits near symmetry between right and left wings with a nominal value of 70° during the first flap cycle, which is close to the value observed by Windes *et al*. [35] during straight descending flight. However, in turning flight they observed a large asymmetry between the two wings, with the wing inside the turn (right) exhibiting a much lower value $\left(40^{\circ }\leq \gamma_{h}\leq 60^{\circ }\right)$, indicating more lateral movement of the wingtip, accompanied by a nominal increase to about 80° for the left wing. The asymmetry was largest at the initiation of the manoeuvre but was reconciled gradually over five flap cycles as the bat settled into the turning-ascending manoeuvre. In the current flight, as the bat initiates the manoeuvre in the second cycle, asymmetry develops between the two wings which keeps increasing during the rest of the recorded fight. During this two flap duration, $\gamma_{h}\;$ tends to 95° and 35° for the left and right wings, respectively. It means that the left wing flaps in a plane which is outward tilted from the long axis of the body (*x_b_*), whereas the right wing flaps much closer to the body. Thus, the nature of the asymmetry observed by Windes *et al*. [49] and Iriarte-Díaz & Swartz [44], that during initiation and into the turn, the wing on the inside of the turn exhibits smaller horizontal stroke plane angles than the wing on the outside of the turn is confirmed in this study as well. This identifies a general trait that the wing inside of a turn flaps closer to the body in the body-horizontal plane when the bat manoeuvres a turn. This particular action reduces the moment arm of the right wing and could be a key factor in generating the roll moment.

Figure 21 also shows the progression of the vertical stroke plane angles during the manoeuvring flight. The current bat flies with progressively increasing vertical angles ($\gamma_{v}$) for both wings, whereas the previous sweeping turn [49] had somewhat stable values for most of the flight time. The magnitude of $\gamma_{v}$ measured in previous studies by Windes *et al*. [35,49] and Sekhar *et al*. [39] for similar-sized bats and by Iriarte-Díaz & Swartz [44] for a much smaller bat, in straight as well as manoeuvring flight, has ranged between 40° and 60°. Whereas, the current bat shows a clear asymmetry between the right and left wings, with the left wing angle increasing from 55° to 74° and the right wing angle from 35° to 67°. During the entire flight, the current bat has a smaller vertical stroke plane angle for the right wing indicating that the wing outside the turn (left wing) consistently flaps in a plane which is steeper with respect to the body-horizontal plane. The difference of approximately 20° between the left and right wing at the start of the recorded flight reduces to about approximately 7° towards the end. Considering that the right turn ascending manoeuvre is initiated at the second stroke of the current flight, the observed asymmetry between the two wings is a common phenomenon between the current and previous sweeping turn [49]. During the initiation of the right turn manoeuvre of a *H. armiger* at the beginning of the flight, Windes *et al*. also observed an asymmetry between the vertical stroke plane angles of the two wings. However, in that sweeping turn, the right wing exhibited a higher vertical stroke plane angle than the left wing (by approx. 10°) as opposed to the current flight. Thus, it is not possible to draw an unequivocal conclusion on the general role of the vertical stroke plane angle in effectuating the turning-ascending manoeuvre except noting that a higher value is more favourable towards thrust production, and as a consequence, the left wing produces larger$\;F_{x,b}$ (figure 16) and contributes to generating a yaw moment.

**Figure 22.**
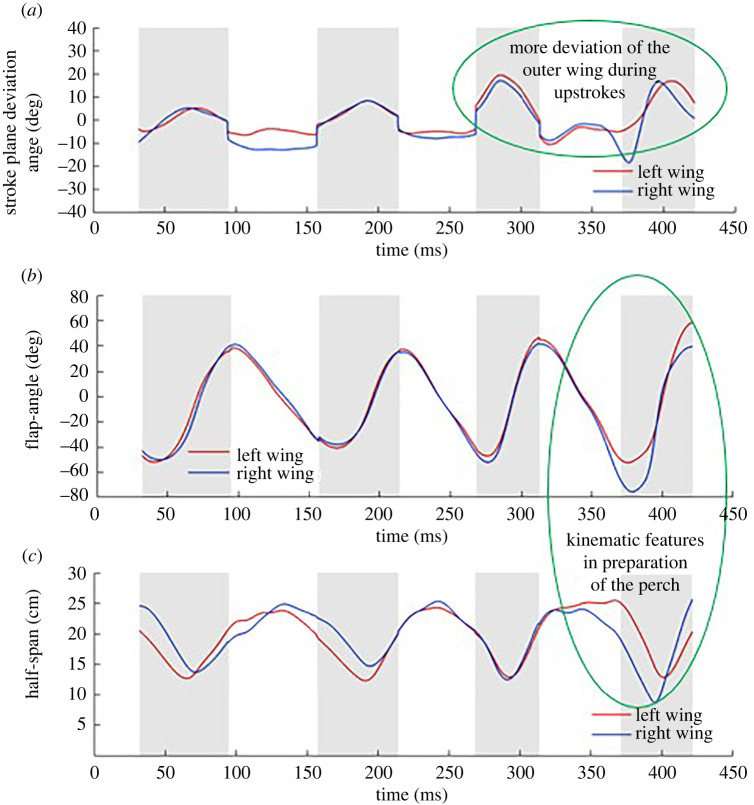
Kinematic features of left and right wings (*a*) stroke plane deviation angle (*b*) flapping angle and amplitude (*c*) half span. Upstrokes are designated by the shaded regions; circled regions indicate kinematic traits associated with the manoeuvre and impending perch.

Additional kinematic markers are shown in figure 22*a–c*. The stroke plane deviation angle is defined as the angle between the actual span-line and its projection onto the stroke plane. Positive values indicate that the wingtip is in front of the stroke plane, while negative values indicate that it is behind the stroke plane. The flap angle is a measure of the flapping amplitude in the stroke plane. The half span represents the instantaneous distance between the wingtip and the respective shoulder joint. While there are drastic differences between the left and the right wing starting at the third downstroke, the observed traits are more likely related to the bat positioning itself for the impending perch on the tunnel ceiling. The flap angle of the right wing excurses dramatically down to −80°, about 20° more than the left wing with its half span dropping below 10 cm, with a much larger than normal stroke plane deviation angle of −20°. During this time, the left wing remains extended out and does not retract towards the body as it normally does at the end of the downstroke. Closer observation of the curvature and ascent angle in figure 8 reveals that although the bat is still climbing, it seems to have straightened its trajectory in preparation for the perch which would require it to execute a 180° somersault. This is why during the third downstroke, there is a steep increase in the ascent angle but the curvature reaches a maximum before reducing to minimal values as the bat rapidly straightens its trajectory. These kinematic traits have a distinct force signature as well—both $F_{x,b}$ and $F_{z,b}$ forces exhibit a secondary peak into the third downstroke. Interestingly, in spite of the large difference in the kinematic traits between the two wings, both wings contribute to the secondary peaks as observed in figures 16 and 17 with the left outer wing having a notably larger contribution to the secondary peak in $F_{x,b}$.

By contrast to the relatively benign differences observed during most of the turning-ascending manoeuvre, Windes *et al*. (Fig. 4 in [49]) found significant consistent differences in the *H. armiger*. They observed that the right wing exhibited higher positive and negative stroke plane deviation angles at mid-upstroke and at end-of-downstroke, respectively, in almost all of the flap cycles during the turning-ascending manoeuvre. They also observed that the right wing had a larger flapping amplitude moving further down in the stroke plane to end the downstroke than the left wing, followed by the observation that the right wingtip also consistently pulled closer to the body than the left wingtip at mid-upstroke.

### 3.9. Energy and power analysis

**Figure 23.**
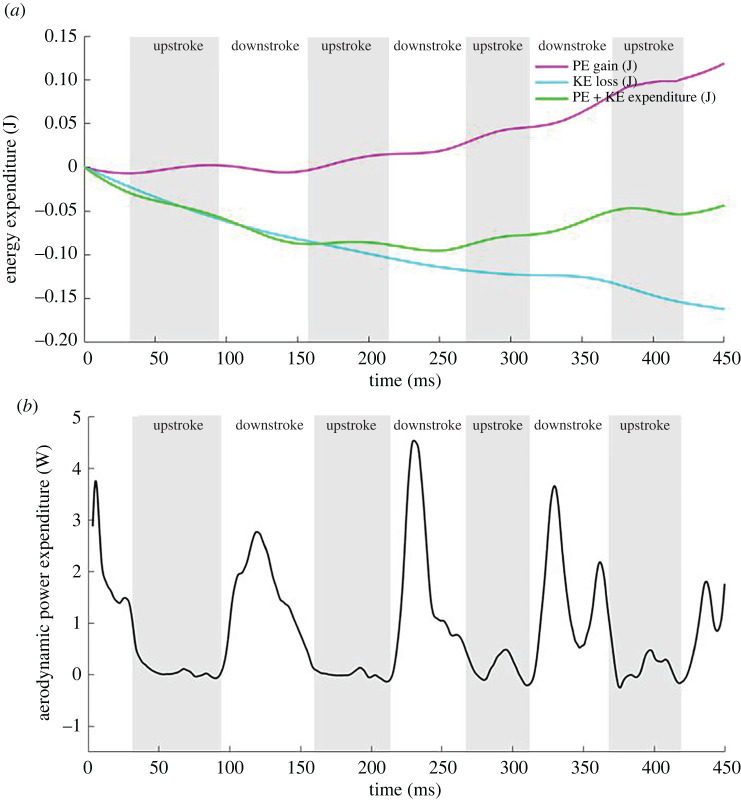
(*a*) Kinetic and potential energy loss and gain and (*b*) power expenditure for the manoeuv ring flight.

While flying, the bat generates power to stay aloft, overcome drag and to manoeuvre through air. For a straight level flight at constant velocity, the basic power required covers two aspects: producing lift to balance its weight and overcoming drag during forward motion. For manoeuvring flight, however, additional aerodynamic power in excess of the base power is needed to turn, climb or accelerate. Figure 23*a* presents the change in potential energy (PE) and kinetic energy (KE) during the flight. The climbing decelerating bat gains PE (energy gain) and loses KE (energy loss). Since the loss in KE is larger than the gain in PE, the bat experiences a net energy loss during the manoeuvre, thus somewhat lowering power requirements. Figure 23*b* presents the aerodynamic power expenditure for the current manoeuvring flight which includes the power expended for gain in PE and power recovered by loss in KE during the manoeuvre. The instantaneous aerodynamic power is obtained from the flow simulation using P=F⋅v on the differential elements of the wing followed by a full surface integration.

Both the ascent and turn are initiated during the second cycle, which is characterized by a sharp increase in aerodynamic power during the second and third downstrokes. While there is some alleviation in power requirements by the bat giving up some of its original KE, the power required for wing articulation and gain in altitude far outweighs that and the net average power for the recorded flight is 0.67 W which is comparable to the power observed during the manoeuvring flight of Windes *et al*. [49] that showed a 94% increase compared with a straight flight.

## 4. Summary and conclusion

We have investigated the kinematics and dynamics of a *H. pratti* performing a turning-ascending manoeuvre. Special attention has been given to the mechanics of executing the manoeuvre and to identify commonalities with the previous investigation of Windes *et al*. [49] in which a *H. armiger* performed a similar manoeuvre under comparable conditions. Both bats used roll and yaw rotations of the body synergistically to redirect the force vector inward to provide the required centripetal force to control the turn. While both yaw and roll rotations can be clearly identified and related to the force moments generated by the wing kinematics, the timing and relative importance of one versus the other varies between the two flights and in general is expected to depend on the initial state of flight entering the turn, the extent of the turn, velocity, etc. For example, the roll moment and roll angle used by the *H. pratti* in the current study are larger than that observed in *H. armiger*, in spite of a similar rate of turn. In generating the roll and yaw moments, the bats use force as well as moment arm asymmetries between the two wings. There is evidence in both flights that the wing inside the turn is drawn closer to the body while executing the turn to reduce the moment arm and accentuate force asymmetries. Force asymmetries are created by introducing phase lags in force generation between the wings and also redirecting force production to different parts of the wing. For example, there is evidence in both flights that the yaw moment is produced by thrust asymmetries on the outer part of the wing.

A common trait noted in both flights was that during the initiation of the manoeuvre, there was a marked increase in the flapping frequency and a shortening of the upstroke compared with the downstroke. Most probably this trait is to power the ascent since a turn can be achieved by introducing force or moment arm asymmetries which may not require a lot more power over and above what is expended by the bat during straight flight. The ascent is characterized by an increase in the lift force in the body-coordinate system. Another observation in both flights was the generation of a small thrust force in the upstroke during the manoeuvre, which is absent in level flight. The thrust peak appeared consistently through the manoeuvre of the *H. armiger* and appeared during the latter half of the flight of the *H. pratti*. A similar peak was also observed by Viswanath *et al*. [64] in a climbing flight of a fruit bat with no lateral manoeuvre. This indicates that the smaller thrust peaks observed during upstrokes are mostly associated with the elevation gain and compensate for the negative component of lift force in the body frame which acts against forward motion on the inclined climbing trajectory of the bat.

The power expended by the *H. pratti* in the current study for the turning and ascending manoeuvre is quite similar to that calculated for the *H. armiger* in Windes *et al*. [49]. In both cases, the average power during the manoeuvre is about 0.67 and 0.66 W, which is approximately twice (approx. 1.91–1.94) the power needed for level flight (0.34 W in Windes *et al*. [49]).

Bat flight has the potential of providing a compelling model for bioinspired MAV designs for agile flight. Thus, it can be beneficial to extract the kinematic and aerodynamic features which bats use to effectuate a certain manoeuvre. This is one of few studies which directly relate kinematics to aerodynamic force generation, which is a critical component for identifying kinematic traits used across different bat species and individuals for a specific manoeuvre. The current study has demonstrated many common traits and features for one manoeuvre, but has also identified kinematic features with markedly different trends; wing kinematic markers such as stroke plane angle, stroke plane deviation angle, flapping amplitude, half span showed many differences between the two flights. Differences were also observed in the relative body orientation with respect to the respective flight trajectories. Further methodical investigation of different manoeuvres across species and individuals will significantly add to a more comprehensive understanding of kinematic asymmetries, aerodynamic forces and power loadings and the interdependence with bat wing morphology.

## Data Availability

Data are available from the Dryad Digital Repository: https://doi.org/10.5061/dryad.mcvdnck2c [65].
